# The Etiology and Pathophysiology Genesis of Benign Prostatic Hyperplasia and Prostate Cancer: A New Perspective

**DOI:** 10.3390/medicines8060030

**Published:** 2021-06-11

**Authors:** Teow J. Phua

**Affiliations:** Molecular Medicine, NSW Health Pathology, John Hunter Hospital, Newcastle, NSW 2305, Australia; Robert.Phua@health.nsw.gov.au

**Keywords:** benign prostatic hyperplasia, prostate cancer, testosterone aging, vascular aging, inflamm-aging, amyloidosis, autophagy, tumorigenesis, prostate stagnation, endothelial dysfunction, inflammation, nitric oxide, oxidative stress, testosterone replacement therapy, aging

## Abstract

Background: The etiology of benign prostatic hyperplasia and prostate cancer are unknown, with ageing being the greatness risk factor. Methods: This new perspective evaluates the available interdisciplinary evidence regarding prostate ageing in terms of the cell biology of regulation and homeostasis, which could explain the timeline of evolutionary cancer biology as degenerative, inflammatory and neoplasm progressions in these multifactorial and heterogeneous prostatic diseases. Results: This prostate ageing degeneration hypothesis encompasses the testosterone-vascular-inflamm-ageing triad, along with the cell biology regulation of amyloidosis and autophagy within an evolutionary tumorigenesis microenvironment. Conclusions: An understanding of these biological processes of prostate ageing can provide potential strategies for early prevention and could contribute to maintaining quality of life for the ageing individual along with substantial medical cost savings.

## 1. Introduction

The etiology and pathophysiology genesis mechanisms of benign prostatic hyperplasia and prostate cancer have not yet been fully elucidated [1,2,3,4,5]. Men’s age is clearly the strongest risk factor [6,7,8,9]. Due to its nature, prostate cancer is considered a heterogeneous disease [10,11,12,13,14,15,16,17].

Benign prostatic hyperplasia is a major health care expenditure in Australia, and this is trending upwards from $5.3million in 2011 to $35.2million in 2018 [18]. Data from the Australian Institute of Health and Welfare predicted that by 2020, 21 out of 100,000 Australian men will die from prostate cancer [19].

This new perspective and overview analyses and evaluates the biological aspects in the accrued interdisciplinary evidence for prostate ageing degeneration, which could provide us with answers to the etiology and pathophysiology genesis mechanisms. These include testosterone ageing, vascular ageing and inflamm-ageing along with amyloidosis and autophagy in cell regulation and homeostasis, as molecular and cellular evidence of prostate ageing in a degenerative multifactorial heterogeneous disease. These new insights into the early evolution of benign prostatic hyperplasia and prostate cancer would allow us to develop strategies for early prevention and maintaining quality of life for the ageing individual along with substantial medical cost savings.

## 2. Testosterone-Vascular-Inflamm-Ageing Triad

The hallmark of testosterone-ageing is declining testosterone levels with age > 40 years, which is clearly demonstrated by the Massachusetts Male Aging Study [20,21]. Testosterone has been shown to regulate the nitric oxide–cyclic guanosine monophosphate pathway and testosterone deficiency is known to induce endothelial dysfunction [22,23], especially with ageing [24]. A preliminary study suggested a family history of prostate cancer may be related to a sharper decline in testosterone level in men over their life course [25]. Endogenous testosterone levels have been shown to be significantly lower in prostate cancer patients [26].

Healthy cellular function, regulation and homeostasis are dependent on the vascular system. The vascular endothelium and nitric oxide-mediated signaling governs the regulation of blood microcirculation [27]. The main hallmark of vascular ageing is endothelial dysfunction, which causes lower peripheral vasodilation [28,29] and is correlated with reduced production of nitric oxide [22,24,30]. The hypoxia inducible factor α expression has been confirmed in ischaemic prostates [31]. Vascular ageing is a chronic vascular inflammatory disease associated with oxidative stress and endothelial dysfunction [32,33,34], which correlate with prostatic hyperplasia carcinogenesis [35,36,37,38,39,40].

Inflamm-ageing is a chronic state of systemic and sterile low-grade inflammation during aging. It is due to the activation of proinflammatory cytokines caused by cell senescence [41,42,43]. Benign prostatic hyperplasia epithelium is enriched with senescent cells [44,45]. The expression of senescence-associated beta-galactosidase in enlarged prostates > 55 g in men with benign prostatic hyperplasia has been detected [46]. Proinflammatory cytokines are elevated with advanced age [47,48] and with testosterone ageing [49,50]. Evidence of an inflammation-specific autoantibody profile and the expression of corresponding autoantigens in prostate tissue have also been detected [51]. Inflammation foci can confound the interpretation of MRI targetable lesions by mimicking prostate cancer, resulting in a 70.5% false-positive rate [52]. Inflamm-ageing increases the oxidative stress level, a key component of chronic inflammation [53] and prostate carcinogenesis [54,55,56,57]. Inflammation correlates to prostate cancer aggressiveness [58,59] and symptomatic benign prostatic hyperplasia [60,61].

Prostate tissue remodeling/degeneration is part of the ageing process, leading to changes in smooth muscle function, prostate growth, enlargement and fibrosis; disrupting prostatic functions [62,63] and with local inflammation being an important contributor [64,65,66,67]. Intact innervation and contractile mechanisms of prostatic smooth muscle are essential for the expulsion of prostatic fluid from the prostate into the ejaculate [68,69], and such innervation is reduced in benign prostatic hyperplasia and ageing groups [70,71,72]. Ageing and hormonal declines are associated with perivascular nitrergic nerves dysfunction, and also with hypertension, diabetes, obesity and cirrhosis [73].Experimental reduction in the androgen level induces stromal remodeling, leading to replacement of smooth muscle cells with fibroblasts or myofibroblasts [74] and in hypoxia, oxidative stress and chronic prostate ischaemia [75]. Symptomatic benign prostatic hyperplasia has been shown to have an increased level of pro-inflammatory prostatic osteopontin [76].

The early protective role of p53 in suppressing inflammation and cancer are strongly associated through the regulation of important cellular activities of the cell cycle of senescence and apoptosis [77,78,79]. The missense mutations in the TP53-gene are found most frequently across all cancer types and give rise to mutant p53 proteins that lose their tumor suppressive activities [80,81,82]. Apoptosis and inflammation play important roles in the control of cell growth and the maintenance of tissue homeostasis, with such disturbances of apoptosis machinery linked to benign prostatic hyperplasia [83]. Cellular senescence is a specialized form of growth arrest and plays a critical role in tumor suppression and aging, with autophagy activated during the process of senescence [84]. Telomere shortening has been demonstrated in benign prostatic hyperplasia, which is associated with prostate epithelial cell senescence [85].

Central to testosterone-vascular-inflamm-ageing triad is the early induction of amyloidosis and autophagy, which play a role in early tumor suppression in terms of the cell regulation pathways, and in their dysregulation in late stages where they act as tumor promoters [86,87,88,89,90]; this correlates to “ageing autophagy” [91,92,93,94]. Short term estrogen reduction using aromatase inhibitor in the adult Wistar male rat alters the prostatic function by reducing nitric oxide availability, inducing amyloid deposition and limiting the differentiation of basal cells through a lobe specific p63-overexpression [95]. Incidentally, these findings can be equated with amyloidosis and autophagy. That is, amyloid deposition and “arrested” basal cells [96,97] equate to amyloidosis as a natural physiological response to stressors of nitric oxide reduction [98,99] and p63overexpression (p53-family) equates to autophagy induction [100].

## 3. Amyloidosis

Currently the pathology of amyloidosis diverges according to opposite viewpoints. These are represented by two reviews as “amyloidoses are a rare disorder” [101] or the ubiquitous “serum amyloid A proteins in “secondary” amyloid disease” [102,103]. Because of the divergent viewpoints, “amyloidosis in prostate” is rarely used as a description, even though amyloid bodies/fibril is found in abundance in the prostate. The use of the term amyloidosis is widely used throughout many publications in relation to many diseases and care must be taken to determine the precursor protein type. The incidental findings of prostatic amyloid transthyretin refers to cardiac amyloidosis [104], as opposed to the serum amyloid A [101,102,103]. Amyloidosis results from the accumulation of pathogenic amyloid, most of which are aggregates of misfolded proteins in a variety of tissues, which interferes with their normal physiology and function in chronic inflammatory diseases [103,105,106,107,108,109,110]. It forms the amyloid senescence cascade hypothesis and is harmful to the non-senescent surrounding cells [111]. “Secondary” amyloidosis due to the serum amyloid A proteins is of lysosomal origin [112].

Amyloidosis is a natural physiological response in mammalian cells and enables cells to store large quantities of proteins and enter a dormant state in response to stressors (e.g., hypoxia, oxidative stress) [98,99]. The family of serum amyloid A proteins encoding genes have been well conserved throughout vertebrate evolution [102,103]. Platelet generated amyloid beta (Aβ) amyloidosis may be more common than currently recognized [113]. In cancer cells in the breast and prostate, the process of amyloidosis induces cells to enter a dormant or resting stage [114] and cell lines studies have indicated that amyloid β oligomers inhibit growth of human cancer cells [115]. During the active periods of prostate cancer there is at least a 500-fold increase in the serum amyloid A level, which declines to normal range in remission [116].

Corpora amylacea (starch-like bodies) and calculi are luminal bodies commonly present in benign prostatic acini [117,118] and in prostate [117,118,119,120,121,122,123,124]; they are also found in approximately 25% of men aged between 20 and 40 years [122]. Amyloid formation by the pro-inflammatory S100A8/A9 proteins has been detected in the ageing prostate [125,126,127,128,129]. Hypoxia and the hypoxia inducible factor 1increases S100A8/A9 expression in prostate cancer [130] and is considered an early carcinogenesis event [131,132,133]. It has been demonstrated that S100A9 promotes prostate cancer cell invasion [134]. The p53 mutants can form amyloid-like structures that accumulate in cells [135]. Pro-inflammatory S100A8/S100A9 proteins with amyloid-forming capacity are found in increased expression levels in many types of cancer, neurodegenerative disorders, inflammatory and autoimmune diseases [127,128]. Patients with glomerulonephritis-associated amyloidosis have higher risk of malignancy [136] and amyloid beta buildup in glioma tumors is a part of the tumor environment [137].

The prostate stagnation hypothesis suggests a possible prostatic accumulation of potentially carcinogenic secretions [138] and fits with the description of a tumorigenesis inflammatory microenvironment [139,140,141,142]. Both prospective reports from the Health Professionals follow-up study cohort based on 8 years and an additional 10 years of follow up, provide the strongest evidence for the a beneficial role of more frequent ejaculation in preventing prostate cancer for men less than 50 years old [143,144]. Similar “stagnation” were shown in middle-aged beagle cohorts, with their prostatic function declining abruptly after 4 years of age [145,146]. Trans rectal ultrasonography studies suggest that the commonly found prostatic calculi may be caused by the obstruction of prostate secretions around enlarged tissues or occlusion by chronic inflammation via benign prostatic hyperplasia [147]. Prostatic secretions expressed by digital rectal massage in 8/10 chronic prostatitis cases showed more signs of prostatic inflammatory aggregates and prominent positive periodic acid–Schiff protein when compared to semen obtained by ejaculation; suggesting the total ejaculate of prostatitis patients contains only a minimal amount of prostate secretions [148]. Regular resistance training exercises and prostatic massage can also reduce the level of proinflammatory markers and improve PSA levels in men with prostate cancer [149].

A 78-year-old man with an enlarged prostate and urinary symptoms who was treated with 10 prostatic-massages combined with antibiotics showed symptom improvement, with the trans rectal ultrasound documenting a reduction in the prostate volume by 52% (63 g to 30 g) [150]. Citric acid secretion studies in 25 men with enlarged prostates, who were given 10 sessions of prostatic massage over 3 to 4 weeks, showed that the hypertrophy receded in almost all cases [151]. Other studies showed symptom improvement for chronic prostatitis, acute urinary retention and lower urinary tract symptoms in patients with repetitive prostatic massage, and with or without antibiotics [152,153,154,155]. The presence of intraluminal inclusions in the prostate cancer tissues promotes remodeling with disruption of the glands’ secretory cycle and drainage function, leading to mechanical trauma, chronic inflammation, and fibrosis development [156]. The prostate corpora amylacea depositions are often a few millimeters in diameter, and can constitute up to a third of the bulk weight of the prostate gland [126].

## 4. Autophagy

Amyloidosis is countered by autophagy and the ubiquitin proteasome system, both of which are major degradation pathways for many disease-associated protein aggregates [105,157]. Autophagy it is a natural regulatory mechanism of the cell that eliminates unnecessary and dysfunctional cellular components to maintain homeostasis [158] and in response to cellular stress [159,160].

Experimental data support a model where autophagy induction as a cytoprotective response promotes cell survival under hypoxia in human prostate stromal cells [161], and decreased autophagy flux in the prostate gland may be implicated in benign prostatic hyperplasia [162]. One of the pivotal contributions of autophagy in immunity is the cell’s autonomous control of inflammation, which represents an anti-inflammatory mechanism [163]. Two natural compounds, oleanolic acid and ursolic acid in low doses, inhibit benign prostatic hyperplasia cell growth by inducing autophagy and reducing the IL-8-axis inflammatory expression in benign prostatic hyperplasia epithelial cells [164]. Autophagy deactivation is associated with severe prostatic inflammation in patients with lower urinary tract symptoms and benign prostatic hyperplasia [165].

The regulatory dynamic of autophagy in cancer metastasis is multifaceted as it plays a suppressive role in early tumors or a promoting role in late stage tumors [86,87,166,167]. Using a histiocytic lymphoma cell line U937 under oxidative stress and DNA damage conditions, it was found that experimental autophagy inhibition induces high cytotoxicity while autophagy induction reduces genotoxicity [159]. Aurora-A kinase over-expression was significantly higher in human prostate cancer specimens than in benign prostatic hyperplasia, and data suggest that aurora-A kinase plays an important role in the suppression of autophagy, which in turn prevents autophagy-induced apoptosis in prostate cancer [168]. Autophagy is deregulated in ageing and human disease [169].

## 5. Evolutionary Tumorigenesis Microenvironment

Evolutionary theory dictates that natural selection is the survival of fittest in the changing environment [170,171]. The prostate ageing degeneration process provides a point of cross-talk between the testosterone-vascular-inflamm-ageing triad, amyloidosis and autophagy, within a prostate stagnation tumorigenesis microenvironment [172]. Together, this tumorigenesis microenvironment and evolutionary biology forms the “evolutionary tumorigenesis microenvironment model”, which could explain the local ecology [173] of degenerative, inflammatory and neoplasm progressions of prostatic diseases, which can span over at least three decades [174]. This could account for a “linear timeline evolutionary pressure” proportionate to gradual natural selection as a slow mutational wave [175] for the emergence of cell subsets’ (distinct phenotypes) survival [176,177,178,179,180,181,182,183,184,185,186], in adapting to the increasingly changing prostate pathophysiology microenvironment [57,58,179,187,188,189,190,191,192]. It also aligns well with the natural progression of the disease and symptom severity during the course of the ageing prostate. Hypoxia localised prostate cancer is associated with elevated rates of chromothripsis, allelic loss of PTEN and shorter telomeres [191].

The timeline of evolutionary biology of prostate ageing-related etiology and pathophysiology genesis takes the form of three phases, in terms of its degenerative, inflammatory and neoplasm progressions:

(i) From about 40 years and onwards is the early period asymptomatic phase at the start of testosterone, vascular and inflamm-ageing, and their effects are mitigated by the prostate being largely functional. However, it is the beginning of nitric oxide down-regulating, oxidative stress, ischaemia hypoxia, chronic inflammation, amyloidosis corpora amylacea, autophagy induction, and remodeling degeneration.
(ii) From about 50 years and onwards is the mid-period mild symptoms phase, which includes the development of lower urinary tract symptoms and benign prostatic hyperplasia [6]. This is due to the incremental prostate ageing degeneration effects of nitric oxide down-regulating, oxidative stress, ischaemia hypoxia, chronic inflammation, amyloidosis corpora amylacea, autophagy induction, and remodeling degeneration
(iii) From about 60 years and onwards is the late period acute symptoms phase, which includes the co-morbidities of benign prostatic hyperplasia, erectile dysfunction, bladder outlet obstruction and adenocarcinoma growth. This is the threshold point at the start of “prostate reprogramming” and the “loss” of cell function, homeostasis and regulation pathways [192,193,194,195,196,197]. It marks the beginning of a prostate stagnation tumorigenesis inflammatory microenvironment with heterogeneous events [17] including inflammation [57,140,141,142,198], genetic aberrations [199,200,201,202,203,204,205], epigenetic dysregulation [206,207,208,209,210], autophagy dysregulation [86,87,89,90,211,212,213,214,215,216] and lysosomal dysfunction [217,218,219,220].

## 6. Prevention

**Figure 1:**
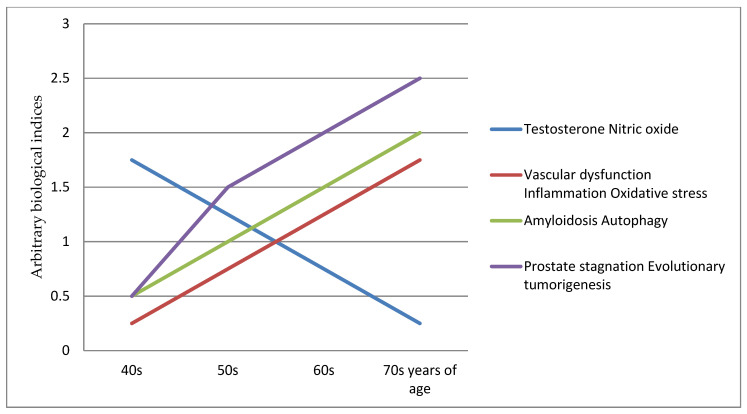
Prostate ageing degeneration hypothesis schematic chart.

This prostate ageing degeneration hypothesis postulates that this triad of testosterone, vascular and inflamm-ageing results in conjoining nitric oxide down-regulating, vascular/endothelial dysfunction and inflammation, with the induction of amyloidosis and autophagy. These are the key etiology and pathophysiology contributors to the prostatic diseases within the evolutionary tumorigenesis microenvironment. It provides a framework for integrating new evidence into a comprehensive concept of a timeline of evolutionary cancer biology of prostate ageing as degenerative, inflammatory and neoplasm progressions of the diseases, for at least a 30 years period (Figure 1). This is a testable hypothesis where biomarkers panel sets can be used to chart the course and range of the ageing degeneration processes.

The future paradigm shift involves an emphasis on prevention as early maintenance of healthy vascular function is necessary to preserve cell function, homeostasis and regulation [166,193], thus prolonging the function of the prostate gland, and delaying/avoiding late stage amyloidosis and autophagy dysregulation [86,87,88,89,90]. Other, potential strategies for ameliorating these biological processes of endothelial dysfunction, oxidative stress and inflammation [221,222,223] could be developed. These should also be complemented consistently with a healthy diet and lifestyle [224,225,226,227].

The key in preventive medicine is to prevent the disease from developing by catching or stopping it early; in this case between the fifth (40s) and before the seventh (60s) decade of life [228,229,230]. A potential three-pronged approach can be explored:

**Testosterone replacement therapy:** long term replacement therapy should be considered to maintain the vascular function; this is a topic of importance that is discussed below as it is an integral part of the prostate ageing degeneration hypothesis.

**Nutraceuticals supplement:** three supplement combinations [231,232,233,234,235,236,237,238,239,240] are necessary to ameliorate the biological processes of endothelial dysfunction (e.g., *l-*citrulline [241,242,243], *l*-arginine [244,245]), oxidative stress and inflammation [246]; publications on this topic are extensive and therefore it is not discussed here.

**Prostate stagnation:** a standard operating procedure using a patented prostate device US8182503B2 could be developed for regular periodic home use for prostate-rectal drainage [155,247,248], in order to modulate the prostate-stagnation tumorigenesis inflammatory microenvironment [139,249]; this is yet to be fully investigated.

Testosterone replacement therapy has been mired in controversy since its introduction in the 1930s up until to the present day [250,251,252,253,254]. Similarly, findings from the Women’s Health Initiative trial of continuous conjugated equine estrogens alone reported two years later, which suggested prevention of coronary heart disease in women who began hormone replacement therapy at age < 60 years and an overall reduction in breast cancer, were largely ignored [255]. This highlights the “window of opportunity and timing” hypothesis, in which the age of starting hormone replacement therapy affects its risk [256] and with “yin-yang” roles [257]. Nitric oxide is one of the most well studied and recognized female estrogen-induced vasodilators [258,259,260,261].

Important health problems in men such as type 2 diabetes, insulin resistance, erectile dysfunction, benign prostatic hyperplasia and depression have been shown to share common pathological processes, such as endothelial dysfunction and inflammation [262], and in numerous testosterone-related concomitant disease and comorbidities [263,264,265,266,267,268,269,270,271,272,273,274,275,276,277,278,279,280,281,282,283,284,285]. Men with low testosterone levels exhibit increases in cardiovascular disease risk markers [286], micro vascular dysfunction [274,287] and these are associated with higher prostate cancer aggressiveness [288]. Both aggressive and metastatic prostate cancer are influenced by metabolic alterations and cardiovascular disease [289], and the progression in hormone naïve prostate carcinomas correlates with low numbers of vascular vessels [290]. In human surgical specimens, there is evidence that local atherosclerosis of the prostatic artery is significantly associated with prostate size [291]. The use of nicorandil, a nitrate derivative to increase the blood flow, reduces the development of prostatic hyperplasia [292]. Sclerotherapy of the internal spermatic veins restores normal supply of testosterone to the prostate solely via its arterial supply, resulting in a significant decrease in prostatic volume and symptoms [293]. Findings from a study suggest that endothelial dysfunction is associated with lower urinary tract symptoms in men [274]. Experimental testosterone deprivation orchiectomy studies showed induced changes to the prostate of rats, and testosterone replacement therapy was effective in reversing such alterations [294]. In two 60-day studies, canine orchiectomy lowered prostate vascularisation [295] and blood volume [296].

Erectile dysfunction is associated with prostate cancer incidence [297] and vascular function. Sleep fragmentation, benign prostate obstruction and nocturnal frequency could decrease sleep-related erections, reflecting the patient’s relevant erectile function [298]. Long term testosterone therapy improves long term blood circulation of penile arteries, penile length and girth, erectile function, and nocturnal penile tumescence and duration [299]. Low androgen status decreased the nitric oxide production and impaired erectile function of rats [300] and electrical penile erection stimulation in mice induced angiogenesis, cell survival and proliferation, and anti-fibrosis signaling pathways [301].

Nitric oxide serves many biological functions [302,303,304]; ageing is frequently associated with *l*-arginine deficiency [305,306] and with the menopausal transition in women [307], as a substrate for nitric oxide synthase. Both oral *l*-citrulline and/or *l*-arginine supplementation increases nitric oxide bioavailability levels in plasma and tissue [241,242,243,244,245,308]. L-arginine restores doxorubicin-induced vascular dysfunction in cancer treatments by attenuating vascular nitric oxide release and apoptosis [231]. Emerging evidence suggests that increasing nitric oxide bioavailability or endothelial nitric oxide synthase activity activates telomerase and delays endothelial cell senescence [309].

A collaborative analysis of the worldwide data on endogenous hormones and prostate cancer risk, found no risk association [310]. In cancer, the two-concentration (biphasic) hypothesis of nitric oxide has determined that low levels of nitric oxide are cancer promoting, while high levels of nitric oxide are protective against cancer [311,312,313,314]. The acquisition of hypoxia-induced malignant phenotypes in tumor cells is impeded by nitric oxide activation of cyclic guanosine monophosphate signaling [315]. Nitric oxide promotes apoptosis and inhibits autophagy in human liver cancer cells [316]. In autophagy, tripartite motif 36 expression is increased in response to androgen and has a prostate cancer suppressive role [317,318,319]. Loss of testosterone impairs anti-tumor neutrophil function [320].

Testosterone replacement therapy itself is able to reduce endothelial dysfunction, oxidative stress and inflammation [49,50,321,322,323,324,325,326,327,328], and is used as treatment for lower urinary tract symptoms and erectile dysfunction [299,329,330,331,332].

## 7. Conclusions

The disease criteria used by the World Health Organization were applies to human biological ageing and it has been found that aging fits the ICD-11 criteria and can be considered a disease; it is included in the extension code for “Ageing-related” (XT9T) in the “Causality” section of the ICD-11 [333].

Tissue degeneration and loss of organ function are features of ageing; conversely, cancer is a state of sustained cellular proliferation and the gain of new functions [42].

Therefore, the most advantageous and best chance strategy is early preventive intervention before tissue damage sets in, and to maintain the vascular function of the ageing prostate gland for as long as possible. Could early, long term testosterone replacement therapy be the Achilles’ heel of prostate cancer? A large preventive trial is warranted to discover the answers to this important question.

## Data Availability

Not applicable.
